# Investigating the Association Between Smoking and Hyposalivation: A Case–Control Analysis

**DOI:** 10.5041/RMMJ.10556

**Published:** 2025-10-31

**Authors:** Ambrose Winnifred Christy, Suresh Kumar Bavesh, Thomas Jones Raja Devathambi, Rajasekaran Thanigainathan

**Affiliations:** Department of Oral Medicine and Radiology, CSI College of Dental Sciences and Research, Madurai, Tamil Nadu, India

**Keywords:** Hyposalivation, saliva, smoking, xerostomia

## Abstract

**Background:**

Xerostomia, or dry mouth, often intensifies oral health problems like dental caries and periodontitis. Smoking is a key factor influencing salivary flow, potentially leading to these issues. This study assesses the prevalence of xerostomia and reduced salivary flow (hyposalivation) among smokers.

**Materials and Methods:**

As case and control groups, the study groups include 150 smokers and 150 healthy non-smokers. A detailed questionnaire was used to collect data on smoking behaviors and symptoms associated with xerostomia. A modified Schirmer test was conducted at 1, 2, and 3-minute intervals to measure unstimulated salivary flow. Descriptive statistics were calculated for age, sex, type, frequency, and duration of smoking. The Mann–Whitney test was done to compare the salivary flow between smokers and non-smokers and to compare smoking parameters with salivary flow. Correlation was also determined for salivary flow with age and smoking parameters.

**Results:**

All the smokers were males, and most were cigarette smokers (86%). Xerostomia symptoms were reported by 19% of smokers and none by non-smokers, which was statistically significant (*P*<0.000). Salivary flow rates at 1, 2, and 3 minutes were significantly lower in smokers than in non-smokers. A comparison between the frequency and duration of smoking and salivary flow yielded statistically significant *P* values of 0.005 and 0.043, respectively. There was a weak negative correlation between age, frequency of smoking, duration of smoking, and salivary flow.

**Conclusion:**

This study found a clear association between long-term smoking and xerostomia, with a notable decrease in unstimulated salivary flow. This highlights the adverse effect of smoking on oral health, which could be used in effective counseling for tobacco cessation.

## INTRODUCTION

Saliva is a vital and complex biological fluid that plays a crucial role in maintaining the health and stability of the oral cavity, aiding in the digestive process, and providing antimicrobial protection. A decreased salivary flow can significantly increase the risk of oral diseases such as periodontitis, dental caries, and infections like candidiasis.^1^–^3^

Xerostomia, or dry mouth, is a subjective condition where the mouth feels persistently dry due to a lack of saliva. This can occur due to several reasons, such as ingesting certain medications, radiation therapy, having medical conditions like diabetes or Sjögren’s syndrome, or simply being dehydrated. Beyond oral health, xerostomia can make everyday activities like eating, speaking, swallowing, and tasting more difficult and uncomfortable.^4^–^6^

Patients with xerostomia may or may not show objective signs of hyposalivation, and these two terms are used independently, with very poor correlation between them.

The salivary flow rate is influenced by several factors, including smoking, a known risk factor that reduces saliva production. Tobacco smoke’s components can cause structural and functional changes in saliva, negatively impacting its protective properties and overall effectiveness in maintaining oral health.^6^

Diagnosing xerostomia and hyposalivation requires using simple diagnostic procedures and eliciting a thorough medical history. Various tests are available to assess the qualitative and quantitative salivary secretion. The modified Schirmer test (MST) is a reliable and effective tool for measuring salivary flow within the oral cavity, providing an objective assessment of dry mouth.^4^,^7^ The present study aims to investigate the prevalence of xerostomia and hyposalivation in smokers and non-smokers, using the MST as a diagnostic tool.

## MATERIALS AND METHODS

Subjects for this cross-sectional observational study were drawn from patients referring to the Department of Oral Medicine and Radiology for routine dental care. Institutional ethical clearance was obtained before the onset of the study (CSICDSR/IEC/0220/2022). A convenience sampling method was employed. The sample size was calculated as 300 according to the following formula: n=N/(1+Ne^2^), where n=number of samples, N=total population, and e=confidence level. A 95% confidence interval was considered. The study population was split equally into two groups: healthy smokers and healthy non-smokers. The study group was composed of apparently healthy individuals who had smoked tobacco at least three times a day for a minimum of six months.

The exclusion criteria were alcohol consumption, use of dentures, a history of radiotherapy, systemic or salivary gland diseases, and those under any drug therapy. These factors were excluded to avoid confounding variables that could affect the results.

### Evaluation of Xerostomia

Six binary questions (Yes/No) adapted from the questionnaires by Fox et al.^5^ and Pai et al.^8^ were used to evaluate patients’ feelings of mouth dryness (Table 1). Patients who responded positively to any one of the questions were classified as having xerostomia. The type of questions receiving a “Yes” determined xerostomic severity (mild, moderate, or severe) and were expected to be an indication of symptom severity. If questions in more than one category received a “Yes,” the higher severity level was recorded for that patient.

**Table 1:** Binary Questions Used for Determining Severity of Xerostomia.

#	Question Asked	Xerostomia Grade Based on “Yes” Answers
1	Do you feel your mouth is dry?	Mild xerostomia
2	Do you sip liquids to aid in swallowing dry food?	

3	Do you feel thirsty very frequently?	Moderate xerostomia
4	Do you have difficulties swallowing any food?	

5	Does your mouth feel dry throughout the day?	Severe xerostomia
6	Do you chew gum/hard candies daily to relieve oral dryness?	

If multiple questions were answered “Yes,” the most severe grade was applied (Severe > Moderate > Mild).

### Evaluation of Hyposalivation using the MST Procedure

The MST assessed unstimulated whole saliva. Following the protocol described by Fontana et al., the test was administered between 10 a.m. and 12 p.m. Participants were instructed to abstain from eating or drinking for 2 hours before the test.^9^ After a short relaxation period, subjects were seated upright in a dental chair. To ensure accurate results, participants were asked to swallow once to clear any excess saliva and then to avoid swallowing during the test. They were also instructed to lift their tongues while the test strip was carefully positioned to prevent accidental wetting. Using cotton pliers, the strip was held vertically with its rounded end placed on the floor of the mouth, either on the right or left side of the lingual frenum. The strip’s color changed to brown upon contact with saliva. Measurements were recorded immediately at 1, 2, and 3-minute intervals. A reading of less than 25 mm at the 3-minute mark was interpreted as indicative of hyposalivation.

### Statistical Analysis

The Mann–Whitney test was used to analyze differences in xerostomia, hyposalivation, and mean salivary flow between smokers and non-smokers. The chi-square test assessed the association between xerostomia and hyposalivation. Descriptive statistics, including means and standard deviations, were calculated. Statistical analysis was performed using the SPSS 13 software. A *P* value of less than 0.05 was deemed statistically significant. Correlations between salivary flow, age, and smoking parameters were also evaluated.

## RESULTS

The study included 150 healthy smokers and 150 healthy non-smokers, who were age-matched, representing the case and control groups, respectively. The total number of participants, mean age of the smokers, and mean salivary flow in both groups are given in detail in Table 2.

**Table 2:** Baseline Characteristics of Smokers and Non-smokers.

Parameter	Total (*n*=300)	Smokers (*n*=150)	Non-smokers (*n*=150)	*P* Value
Age in years, mean (SD)	36.3 (13.6)	38.8 (13.7)	33.9 (13.0)	Not calculated*

Male sex, number (%)	295 (98.3)	150 (100)	145 (96.7)	Not calculated†
Female sex, number (%)	5 (1.7)	0 (0)	5 (3.3)	
Mean salivary flow, mL (SD)‡				

At 1 minute		13.1	21.8	<0.001

At 2 minutes		19.3	29.0	<0.001

At 3 minutes		25.1	32.9	<0.001

* Both groups fell within the accepted frequency distribution; hence, *P* values were not calculated.

† Due to the low number of female participants, sex was not correlated with salivary flow and hence no *P* value was calculated.

‡ The Spearman Rank Correlation was used for salivary flow and assessment of the relationship between variables.

Cigarette smoking was the predominant form of tobacco use among smokers (86%), whereas 16 participants smoked both cigarettes and bidis (a traditional form of cigarette consisting of sun-dried tobacco flakes rolled in a tendu leaf); 11 cigarette smokers also used smokeless tobacco products occasionally. The mean frequency of smoking per day was 6.43 times, and the duration of smoking was around 12.95 years in the study group. Additionally, smokers with higher mean smoking frequency and longer duration of smoking habit experienced xerostomia more than did other smokers (Table 3).

**Table 3:** Age, Frequency, and Duration of Smoking in Smokers With and Without Xerostomia.

Parameters	Xerostomia	No xerostomia	*P* value
Distribution, *n*	29	121	0.713
Age in years, mean (SD)	39.7 (12.8)	38.8 (14.0)	0.710
Frequency of smoking per day, mean (SD)	7.6 (3.5)	6.1 (4.7)	0.005
Duration of smoking in years, mean (SD)	15.1 (10.4)	12.5 (11.1)	0.043

Values calculated using the Mann–Whitney *U* test

SD, standard deviation.

When comparing salivary flow between the smoking and non-smoking groups at three different time points (1, 2, and 3 minutes), smokers showed a significantly lower salivary flow (*P*<0.000) at all three points (Table 2).

A weak negative correlation was found between age, smoking frequency, duration of smoking, and salivary flow at all time points in the smokers and non-smokers groups, which was statistically significant (*P*<0.05) (Table 4, Table 5).

**Table 4:** Correlation of Age with Salivary Flow in Smokers and Non-smokers.

Salivary Flow Time Point	Smokers (*n*=150) *r* (*P*)	Non-smokers (*n*=150) *r* (*P*)
1 minute	−0.188 (0.021)	−0.279 (<0.001)
2 minutes	−0.223 (0.005)	−0.352 (<0.001)
3 minutes	−0.163 (0.046)	−0.330 (<0.001)

The Spearman rank correlation was used for salivary flow.

*P*, *P* values; *r*, Pearson correlation coefficients.

**Table 5:** Correlation of Smoking Frequency and Duration with Salivary Flow in Smokers.

Variable	Salivary Flow Time Point	Smokers (*n*=150) *r* (*P*)
Smoking frequency/day	1 minute	−0.199 (0.015)
	2 minutes	−0.289 (<0.001)
	3 minutes	−0.258 (0.001)

Smoking duration (years)	1 minute	−0.153 (0.062)
	2 minutes	−0.262 (0.001)
	3 minutes	−0.235 (0.004)

*P*, *P* values; *r*, Pearson correlation coefficients.

The Spearman Rank Correlation was used for salivary flow.

Sex-based correlation analysis was not feasible due to the very small number of female participants (5 non-smokers out of 300), hence it could not be reliably assessed.

## DISCUSSION

Saliva is considered a principal defense factor of the oral cavity, which is vital in maintaining oral health. Salivary secretion is closely linked to the state of hydration and is influenced by systemic diseases, medications, and lifestyle habits.^10^ Among these, smoking is known to affect the sensitivity of taste receptors, potentially reducing the salivary reflex. In the present study, subjective sensation of xerostomia was observed in 19% of smokers, while none of the non-smokers exhibited symptoms. These findings align with previous research, such as Dyasanoor and Saddu, who reported a xerostomia prevalence of 37% among smokers and 13% among non-smokers.^3^

The MST, used to measure unstimulated salivary flow, demonstrated its reliability as a practical and non-invasive tool in clinical settings. Consistent with the current findings, other studies have reported MST values below 25 mm at 3 minutes, indicative of hyposalivation, and values below 15 mm suggestive of severe xerostomia. This aligns with Chen et al.’s findings that MST is a valuable diagnostic tool for detecting reduced salivary function.^7^

The average salivary flow, measured using the MST, was significantly lower in smokers than in non-smokers at all time points. At 3 minutes, the difference in mean MST values among smokers and non-smokers was around 7.6 mm, indicating a significant reduction in salivary flow due to smoking (*P*<0.000). This highlights the negative impact of tobacco use on salivary gland function, supporting the notion that smoking is a substantial risk factor for xerostomia and hyposalivation. This study also accounted for age-related variations, as salivary function can decline with aging due to parenchymal atrophy. In addition to the age-related degenerative changes, smoking also negatively influences salivation, which was seen in our study. The correlation between age and salivary flow revealed a weak but significant negative association in both groups, with older participants exhibiting lower flow rates.

Furthermore, smoking frequency and duration were found to exacerbate reductions in salivary flow. Participants with xerostomia smoked more than those without xerostomia, which was statistically significant.

Studies have suggested that smoking not only reduces salivary flow but also affects the composition of saliva, potentially increasing the risk for oral conditions such as cervical caries and periodontal disease.^11^ The direct exposure of oral tissues to tobacco components may result in long-term degenerative changes in salivary glands, further decreasing secretion over time. While some initial increases in salivary flow may occur as a reflex response to nicotine, prolonged smoking often leads to desensitization of taste receptors, thereby depressing salivary reflexes. This effect may explain the lower MST values observed among smokers in the present study.^3^,^12^

Since salivary flow assessments can help identify at-risk individuals, regular screening for xerostomia and hyposalivation in smokers is recommended, especially for those reporting dry mouth symptoms.

Limitations of the study included a convenience sampling method, and a small sample size. Furthermore, the association between smoking-induced oral conditions and salivary secretion was not evaluated.

## CONCLUSION

The study’s findings emphasize the need for early intervention in individuals with smoking-related salivary gland dysfunction to prevent complications associated with dry mouth. Hyposalivation in smokers can be considered as one of the harmful effects of smoking, and its negative clinical indicators on oral health can be effectively used in tobacco cessation. Consequently, dental practitioners should consider comprehensive salivary evaluations and incorporate preventive strategies to manage dry mouth symptoms in smokers.

## Abbreviations

CSICDSR/IEC: CSI College of Dental Sciences and Research/Institutional Ethical Committee
MST: modified Shirmer test.
